# Residual potential at the epicardial left atrium after conventional left atrial posterior wall isolation for persistent atrial fibrillation: A case report

**DOI:** 10.1002/joa3.12365

**Published:** 2020-05-26

**Authors:** Tomoyuki Arai, Seiji Fukamizu, Takeshi Kitamura, Rintaro Hojo

**Affiliations:** ^1^ Department of Cardiology Tokyo Metropolitan Hiroo Hospital Tokyo Japan

Pulmonary vein isolation (PVI) has become the standard treatment for atrial fibrillation (AF) and some studies have reported the benefits of additional posterior wall isolation (PWI) after PVI for persistent AF, which they had confirmed by meta‐analysis.^1^ However, a previous study reported that PWI in addition to PVI did not improve the recurrence of AF.^2^ Furthermore, PWI with additional ablation within the box lesion from 41% to 55% after roof and inferior line ablation has been reported.^3^ However, the epicardial state after additional ablation within the box was unclear.

A 78‐year‐old man visited our hospital complaining of shortness of breath. He had AF recurrence after PVI for paroxysmal AF. Transthoracic echocardiography showed mild pericardial effusion. Written informed consent was obtained from the patient. He underwent a second session for recurrent persistent AF.

We created a LA voltage map by inserting a 20‐electrode mapping catheter (Pentaray; Biosense Webster) via a transseptal approach and using a 3‐dimensional mapping system (CARTO; Biosense Webster). No PV reconduction was recorded, and additional PWI was undertaken (Figure 1A). Roof line ablation was performed during pacing from the right atrium (RA). Target ablation index (AI) was 450 at 40W for contact force–sensing irrigated ablation catheter (Thermo‐Cool Smarttouch SF, Biosense Webster), with the mapping catheter positioned on the PW. We confirmed that the earliest potential site of Pentaray at PW changed from the roof to the inferior side during roof line ablation. Inferior line ablation was performed during pacing from RA and AI was 400 at 30 W for contact force–sensing irrigated ablation catheter. However, PWI was incomplete for the Pentaray positioned on the PW. Therefore, we recreated the LA voltage map which confirmed residual potential within the box. The activation map showed propagation into the PW from the inferior side. Additional ablation within the box was performed at 30 W (AI 400). However, PWI was incomplete (Figure 1B). AF with isoproterenol (ISP) triggered at PW was induced repeatedly. While further ablation was considered, we created an epicardial map to evaluate epicardial state because it was necessary to deal with preoperative pericardial effusion.

**FIGURE 1:**
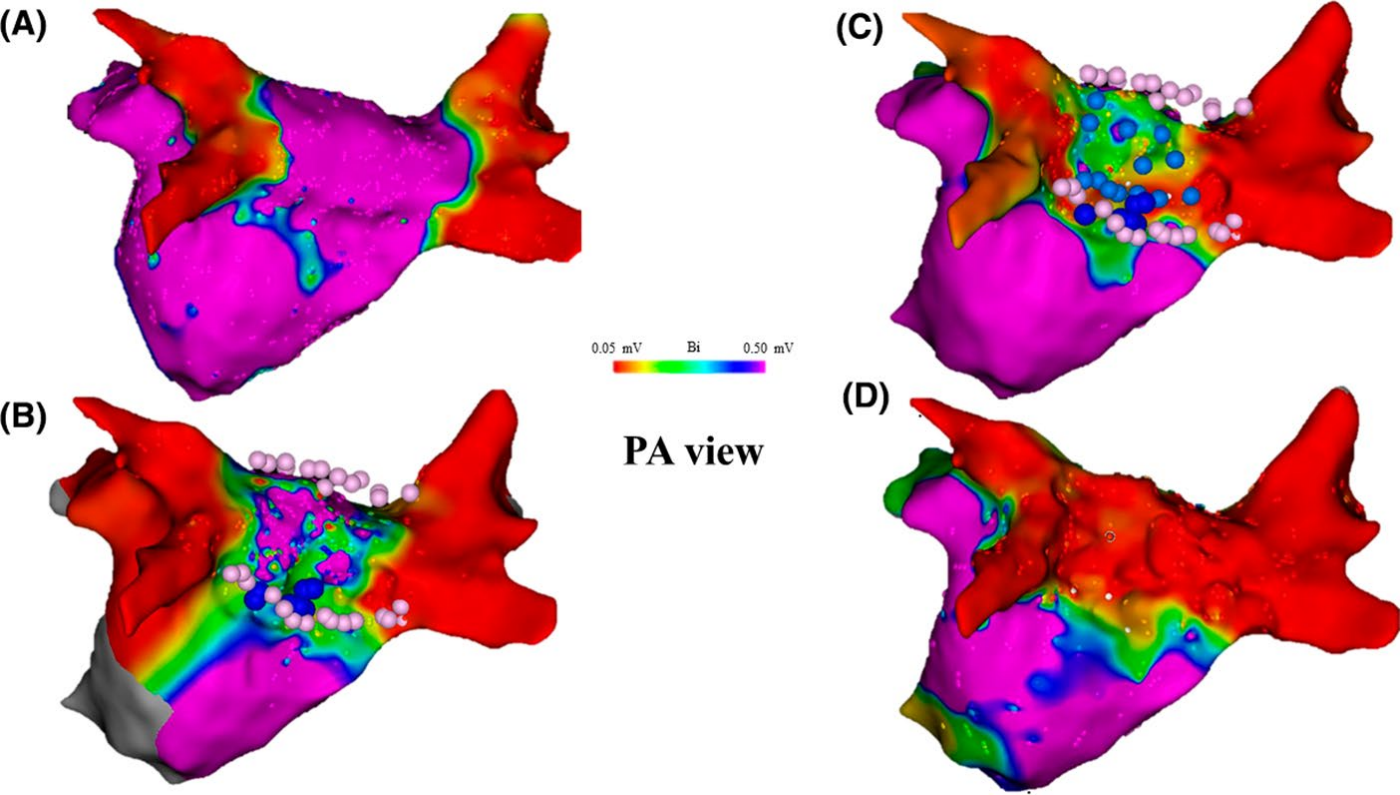
Time course of the left atrial (LA) endocardial voltage map. A, LA voltage map obtained during pacing from the right atrium shows that no pulmonary veins exhibited reconduction. B, The LA voltage map after roof and inferior line ablation showed incomplete posterior wall isolation (PWI) (pink points). Additional ablation was performed within the box. However, PWI was still incomplete (blue points). C, After epicardial mapping, further additional high‐power (50 W) ablation was performed within the PW (light blue points). D, The high‐output pacing could not be captured within the box area and PWI was considered complete. PA, posteroanterior

We confirmed coronary artery flow by coronary angiography. A steerable sheath was inserted via a subxiphoid approach and an epicardial PW voltage map during pacing from the RA was created using a 10‐electrode mapping catheter (Deca Nav; Biosense Webster). The LA voltage map showed preserved potentials at the PW from the epicardial side. The epicardial activation map showed that propagation broke into the PW from the inferior side, like in the endocardial activation map. The thresholds were 6.5 mA (2.0 ms) on the endocardial side (determined using Pentaray) and 1.4 mA (2.0 ms) on the epicardial side (determined using Deca Nav). Ablation was not attempted at the epicardial side to avoid esophageal injury. Stimulation with 20 mA (2.0 ms) was carried out from the endocardium side via contact force–sensing irrigated ablation catheter, and the captured sites were ablated at 50 W/15 s (AI 450) monitoring esophagus temperature (Figure 1C). The high‐output pacing could not capture the entire box area and PWI was complete (Figure 1D). The endocardial LA voltage map indicated the disappearance of potential at the PW (Figure 2A). However, the epicardial voltage map revealed residual potential within the box (Figure 2B). High‐output pacing via contact force–sensing irrigated ablation catheter with endocardial box area could not be captured by the Deca Nav on the epicardial side (Figure 2C). The session ended because of the disappearance of potential at the PW on the endocardial side and lack of AF inducibility with ISP infusion. Postoperatively, the patient continued to take antiarrhythmic drugs and maintained sinus rhythm during the 9 months follow‐up period.

**FIGURE 2:**
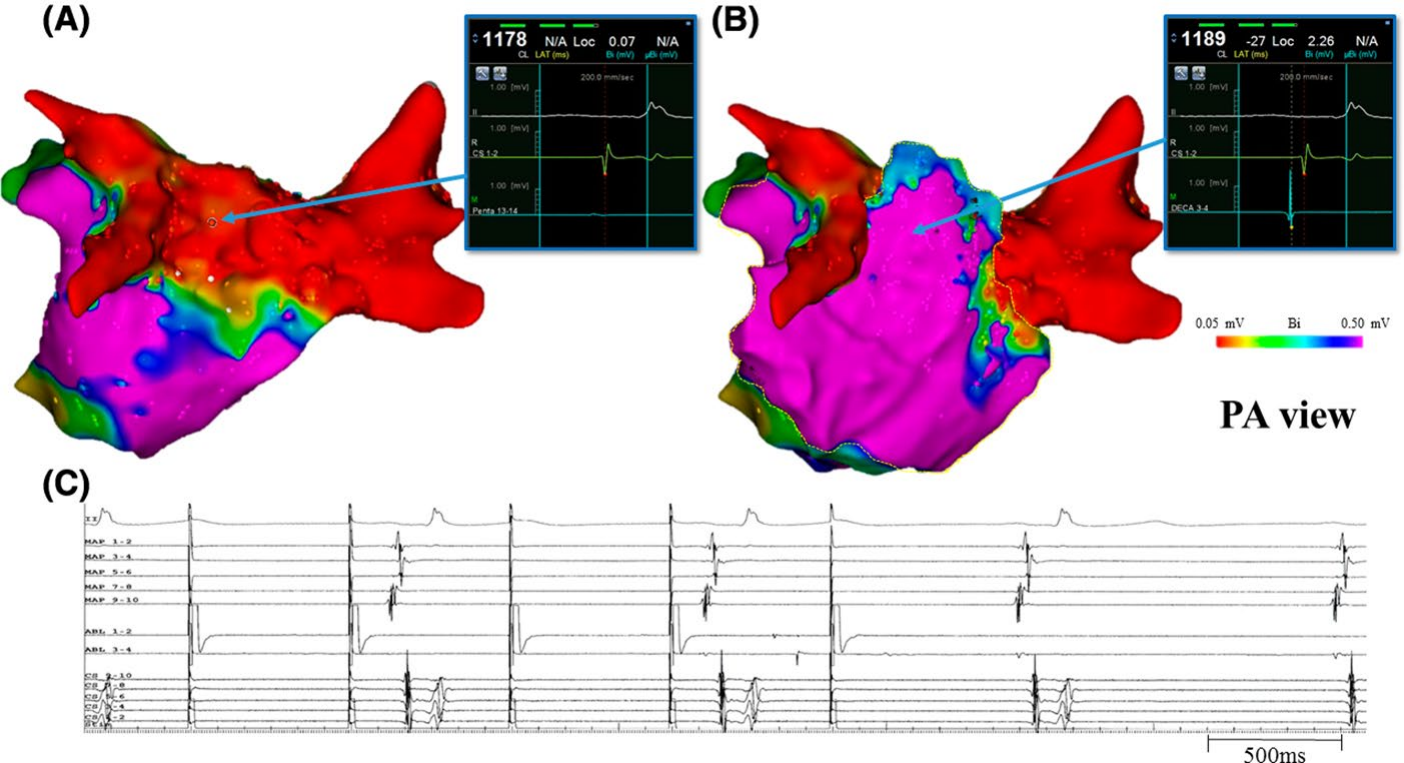
Left atrial voltage maps of the endocardium and epicardium were compared after completion of conventional posterior wall isolation and intracardiac electrocardiogram during high‐output pacing from the endocardium. A, The voltage amplitude was 0.07 mV on the endocardial side recorded by the Pentaray. B, The voltage amplitude was 2.26 mV on the epicardial side recorded by the Deca Nav. C, High‐output pacing via contact force–sensing irrigated ablation catheter within endocardial box area could not be captured. MAP, 10‐electrode mapping catheter (Deca Nav); ABL, contact force–sensing irrigated ablation catheter (Thermo‐Cool Smarttouch SF); CS, coronary sinus; PA, posteroanterior

The endpoint of PWI has been defined as the disappearance of potential at the PW on the endocardial side. However, when high‐output pacing from endocardium was not captured and PWI in the endocardial side was complete, there have never been evaluated the epicardial condition. Despite complete PWI, potential at the PW was detected on the epicardial side in the present case. This suggests that transmural lesions extending to the epicardium at the PW might not always form in conventional PWI.

Jiang et al analyzed endocardial and epicardial voltage maps from 18 patients before and after ablation for AF. Transmural lesions extending to the epicardium were confirmed in six of nine patients who underwent PWI and the remaining three exhibited residual potential in the epicardium.^4^ Nontransmural lesions may cause PW reconduction via the epicardium, increasing the risk of AF recurrence. In the present case, high‐output pacing from the contact force–sensing irrigated ablation catheter was not captured from the endocardium, but potential remained in the epicardium. It has been suggested that transmural lesions could not be achieved by ablation from the endocardium alone. We did not attempt ablation at the epicardial side because of the risk of esophageal injury. Some studies have reported that using an intrapericardial balloon and catheter for mechanical displacement of the esophagus enable safe ablation from the epicardium and increase chance of transmural isolation extending to the epicardium.^4^, ^5^ The contribution of transmural lesion formation to the prevention of AF recurrence is unclear and further study is needed.

In conclusion, PWI using pace and ablate technique may not be able to create transmural lesions extending to the epicardium. Nontransmural lesions might lead to PW reconduction with possible AF recurrence.

## CONFLICT OF INTEREST

None.
